# Cluster-derived immunometabolic profiles: association with glycemic dysregulation in elderly postmenopausal women

**DOI:** 10.1007/s12020-026-04762-w

**Published:** 2026-09-09

**Authors:** Ana Cristina Lira de Menezes, Ingrid Almeida Maciel, Eduarda Caroline Oliveira Barros, Nailza Maestá, Steven S. Witkin, Fábio Lera Orsatti, Cláudio Lera Orsatti

**Affiliations:** 1 Biochemistry – Immunology Research Group – GPB-I, Department Health Science, Oeste Paulista University - UNOESTE, Jaú, SP Brazil; 2 Department of Obstetrics and Gynecology, Weill Cornell Medicine, New York, NY USA; 3 Department of Infectious Diseases and Parasitology, Division of Virology, University of Sao Paulo Faculty of Medicine, Sao Paulo, Brazil; 4 Applied Physiology, Nutrition and Exercise Research Group - PhyNEr, Institute of Health Sciences, Federal University of Triangulo Mineiro - UFTM, Uberaba, Minas Gerais Brazil

**Keywords:** Vitamin D, Insulin resistance, Interleukin-10, Elderly women, Inflammaging

## Abstract

**Purpose:**

To identify variations in immunometabolic profiles in elderly postmenopausal women using a data-driven clustering approach and to examine differences in insulin resistance (HOMA-IR) and glycemic control (HbA1c) across the derived profiles.

**Methods:**

This cross-sectional study included 132 women with a mean age of 70.8 years. Unsupervised hierarchical cluster analysis (Ward.D2 method, squared Euclidean distance) was performed using twelve variables spanning demographic, hemodynamic, metabolic, inflammatory, and vitamin D-related domains. HbA1c and HOMA-IR were compared across clusters; HOMA-IR was not fully independent of cluster derivation because fasting glucose contributes to its calculation. Between-cluster differences were assessed using Kruskal–Wallis tests or one-way ANOVA. DSCF post-hoc tests and epsilon-squared (ε²) effect sizes were used for non-parametric comparisons.

**Results:**

Three cluster-derived profiles were identified (Cluster 1, *n* = 25; Cluster 2, *n* = 50; Cluster 3, *n* = 57). Cluster 1 was characterized by lower IL-10 levels and a less favorable lipid profile, while blood pressure was similarly elevated in Clusters 1 and 2 relative to Cluster 3. Cluster 2 showed an overall intermediate profile, with the lowest vitamin D concentration as its most distinctive feature. Cluster 3 exhibited lower blood pressure, higher IL-10 levels, and a more favorable lipid profile. When examining these additional glycemic measures, HbA1c showed a graded increase across clusters (*p* < 0.05 for all pairwise comparisons), whereas HOMA-IR differed between Cluster 3 and Clusters 1 and 2 but did not differ significantly between Clusters 1 and 2.

**Conclusion:**

The differentiation of elderly postmenopausal women into three data-driven immunometabolic profiles illustrated substantial heterogeneity. These findings suggest that HbA1c may provide complementary information to fasting-based indices when characterizing immunometabolic heterogeneity in elderly postmenopausal women.

## Introduction

Cardiometabolic risk increases substantially after menopause, with older women presenting higher prevalence of dyslipidemia, impaired glucose regulation, and hypertension. These metabolic alterations are associated with hormonal changes accompanying ovarian aging and with age-related metabolic remodeling, which together contribute to unfavorable changes in cardiometabolic health [1, 2]. As a consequence, postmenopausal women represent a population at increased risk for metabolic disorders and cardiovascular disease.

Despite this increased risk, metabolic deterioration during aging does not occur uniformly across individuals. Women of similar chronological age often exhibit markedly different metabolic and physiological profiles, suggesting that aging follows heterogeneous biological trajectories rather than a single linear pathway [3, 4]. This heterogeneity implies that individuals may cluster into distinct multivariate profiles reflecting different configurations of metabolic regulation and cardiometabolic risk [5, 6].

Multiple biological systems appear to contribute to this heterogeneity. Chronic low-grade inflammation, frequently described as inflammaging, has been implicated in metabolic dysfunction associated with aging, including impaired insulin signaling, adipose tissue dysregulation, and cardiometabolic disease [7–10]. In addition to inflammatory processes, micronutrient-related pathways may also modulate metabolic regulation. Vitamin D, for example, participates in immune regulation and has been associated with metabolic and cardiovascular health outcomes in aging populations [2, 11]. These observations suggest that interactions across inflammatory, metabolic, and nutritional domains may contribute to the emergence of distinct immunometabolic profiles in older adults [12, 13].

Among the metabolic consequences of these interactions, resistance to insulin action represents a key mechanism linking metabolic dysfunction to cardiometabolic disease [14, 15]. Insulin resistance contributes to impaired glucose regulation and can be estimated using fasting-based indices such as the Homeostatic Model Assessment of Insulin Resistance (HOMA-IR), whereas glycated hemoglobin (HbA1c) reflects longer-term glycemic exposure [16]. However, these aspects of glucose homeostasis vary substantially among individuals [4], raising the possibility that distinct immunometabolic profiles may also differ in their glycemic characteristics.

Most studies investigating cardiometabolic health in aging populations evaluate biomarkers individually using conventional statistical approaches. Although informative, these strategies may fail to capture complex interactions among inflammatory, metabolic, and nutritional pathways that jointly influence physiological regulation [17]. In contrast, unsupervised multivariate approaches such as cluster analysis allow the identification of data-driven profiles by grouping individuals based on similarity across multiple variables, without assuming predefined biological categories [18, 19]. Previous studies have shown that clustering approaches can reveal distinct metabolic phenotypes and clinically meaningful cardiometabolic risk profiles in heterogeneous populations [5, 6, 20, 21].

It remains unclear whether integrated immunometabolic variables can identify distinct data-driven profiles in postmenopausal women and whether these profiles differ in additional measures of glycemic regulation, particularly HOMA-IR and HbA1c. Therefore, the present study aimed to identify immunometabolic profiles in elderly postmenopausal women using an unsupervised cluster analysis approach based on inflammatory cytokine levels, vitamin D status, age, and cardiometabolic markers. HOMA-IR and HbA1c were intentionally excluded as direct clustering variables, whereas fasting glucose was retained as part of the metabolic characterization used to derive the clusters. After identifying these clusters, we examined differences in HOMA-IR and HbA1c across the resulting profiles as measures of fasting-state insulin resistance and longer-term glycemic exposure, respectively.

## Methods

### Study design and sample selection

This cross-sectional investigation utilized a cohort of 132 volunteers seen at the School of Medicine of Jaú - UNOESTE. All clinical assessments were performed at the Multiuser Laboratory of Biotechnology in Health. This study followed the ethical principles of the Declaration of Helsinki. Written informed consent was obtained from all participants, and the protocol was approved by the Institutional Ethics Committee (number: 7128500).

The study cohort consisted of 132 women who met the eligibility criteria during the recruitment period. This sample size was considered adequate for exploratory multivariate analyses, including hierarchical clustering, based on previous applications in metabolic research [19]. The clustering solution was evaluated based on internal structure (e.g., silhouette index) and interpretability of the resulting profiles. Inclusion criteria followed a subset of elderly women and conformed to the NICE (2017) guidelines: women whose last menstrual period occurred at least 14 years prior, were aged over 60 years, and were either users or non-users of hormone therapy [22]. Exclusion criteria included current or past use of vitamin D supplements within the last 6 months, use of immunosuppressive or anti-inflammatory medications (including systemic corticosteroids), diagnosed autoimmune diseases, active neoplasms or alcohol or drug abuse.

### Clinical and anthropometric data collection

During the interview, clinical information such as age, age at menopause, duration of menopause, parity, current smoking status, history of chronic diseases (hypertension, diabetes, cardiovascular diseases), physical activity practice, blood pressure, weight, and height were collected. Blood pressure was measured in the right arm, with the forearm supported at heart level, palm facing up, using a standard aneroid sphygmomanometer, with the patient seated. Smokers were defined as those who smoked daily, regardless of quantity. Physically active women were considered those who engaged in moderate-intensity aerobic physical exercise for at least 30 min five days a week (150 min/week), or resistance exercises three times a week. The following anthropometric data were obtained: weight, height, body mass index (BMI = weight/height²), and waist circumference. Weight was measured using an electronic anthropometric scale (Filizola®, Brazil), with a capacity of 150 kg and accuracy of 0.1 kg. Height was measured with the patient barefoot, arms alongside the body, and gaze on the horizontal plane. Waist circumference was measured between the last rib and the anterosuperior iliac crest, during expiration. It was considered increased when > 88 cm.

### Laboratory analyses

Twenty mL of blood were collected by venipuncture into both EDTA-containing tubes and dry tubes, for the evaluation of lipid profile, glycemic profile, hormonal profile, cytokines and vitamin D levels. Analyses were performed at the BioHealth Lab at UNOESTE. For the lipid and glycemic profile, total cholesterol (TC), HDL, LDL, triglycerides (TG), and fasting glucose were measured. The tests were processed on an automatic biochemical analyzer RAXT (Technicon®, USA), using a colorimetric method with commercial reagents (Sera-Pak®, Bayer, USA). The method was linear up to 800 mg/dL for TG and 900 mg/dL for TC. LDL was calculated using the formula: LDL = TC – (HDL + TG/5), valid only with TG < 400 mg/dL. Values considered were: TC < 200 mg/dL, HDL > 40 mg/dL, LDL < 100 mg/dL, TG < 150 mg/dL, and fasting glucose ≥ 100 mg/dL was considered elevated [23].

Parathyroid hormone (PTH) and insulin were quantified by ELISA (Quantikine Kit, R&D Systems, Minneapolis, MN, USA) according to manufacturer instructions, with an analytical sensitivity of 1.1 pg/mL for PTH and with an analytical sensitivity of 0.5 μIU/mL for insulin. Insulin resistance (IR) was assessed using the HOMA-IR index, calculated as insulin (μIU/mL) × glucose (mg/dL)/405. The cutoff value of HOMA-IR > 2.7 was adopted according to the Brazilian reference proposed by Geloneze *et al* [24] for the identification of insulin resistance in adult populations. However, because evidence suggests that optimal HOMA-IR thresholds vary according to age and sex, particularly among women, this value was used in the present study solely as a descriptive reference and not as a diagnostic criterion for elderly participants. Importantly, HOMA-IR was analyzed as a continuous variable and was not included in the clustering procedure.

From the obtained serum, 500 μL aliquots were separated and stored at −20 °C until the analysis of 25(OH)D levels, performed by ELISA (R&D® Systems Kit, USA), with a sensitivity of 2.8 ng/mL. Classification was sufficient (≥ 30 ng/mL), insufficient (21–29 ng/mL), and deficient (< 20 ng/mL).

TNF-α, IL-6, and IL-10 were assayed using commercial ELISA kits (High Sensitivity Human TNF, High Sensitivity Human IL-6, High Sensitivity Human IL-10, Quantikine Kits, R&D Systems, Minneapolis, MN, USA), according to manufacturer instructions. All measurements were performed at the same time to avoid inter-assay variation. Intra-assay coefficients of variation were < 7%. The analytical high sensitivity was 0.19 pg/ml for TNF-α, 0.11 pg/ml for IL-6, and 0.17 pg/ml for IL-10.

### Statistical analysis

Continuous variables were assessed for normality using the Shapiro–Wilk test and for homogeneity of variances using Levene’s test. Normally distributed data are expressed as mean ± standard deviation, while non-normally distributed variables are presented as median and interquartile range (IQR). Categorical variables are shown as frequencies and percentages and were analyzed using the chi-square test (χ²) or Fisher’s exact test, as appropriate. No missing data were identified in the final analytical dataset; therefore, all 132 participants were retained in all analyses, and no imputation procedures or complete-case exclusions due to missing values were required.

To identify distinct immunometabolic profiles, an unsupervised hierarchical cluster analysis was performed. This exploratory approach organizes individuals into a dendrogram based on their multidimensional biochemical similarity. Variables included in the clustering procedure were selected a priori based on their relevance to immunometabolic regulation and biological aging. The final model incorporated age, systolic and diastolic blood pressure, fasting glucose, lipid profile (total cholesterol, HDL-c, LDL-c, and triglycerides), inflammatory markers (IL-6, TNF-α, and IL-10), and vitamin D status. Age and blood pressure variables were included because they represent fundamental dimensions of senescence and cardiovascular adaptation in late postmenopausal women.

To reduce redundancy, variables representing overlapping physiological domains were not simultaneously included. HbA1c and HOMA-IR were intentionally excluded as direct clustering variables and were subsequently compared across the derived clusters as additional glycemic markers. Fasting glucose was retained as part of the multidimensional metabolic characterization used to derive the clusters. Therefore, HbA1c can be interpreted as a glycemic marker external to the clustering procedure, whereas HOMA-IR was not considered fully independent of cluster derivation because fasting glucose contributes to its calculation.

All variables were standardized using z-scores prior to clustering to ensure equal contribution to the distance metric. Hierarchical agglomerative clustering was performed using Ward’s minimum variance method (Ward.D2) with squared Euclidean distance. The number of clusters was determined based on dendrogram inspection, fusion distances, the elbow method, and silhouette analysis. A three-cluster solution was selected because it provided the best balance between cohesion and separation (mean silhouette = 0.408), compared with two-cluster (0.246) and four-cluster (0.332) solutions, and yielded biologically interpretable profiles.

Cluster stability was assessed using bootstrap resampling with 1000 iterations in R. For each bootstrap sample, the same hierarchical clustering strategy was repeated, and the similarity between the original clusters and the most similar bootstrap-derived clusters was quantified using the Jaccard similarity coefficient. Mean Jaccard coefficients were calculated for each cluster as indices of cluster stability.

Sensitivity analyses were performed to evaluate the influence of variables with the greatest contribution to cluster separation. The clustering procedure was repeated after sequential exclusion of systolic blood pressure (SBP), IL-10, and both SBP and IL-10. Additionally, fasting glucose was excluded in a separate sensitivity analysis because it contributes to the calculation of HOMA-IR. For each sensitivity model, the clustering structure was reassessed using silhouette coefficients and bootstrap-derived Jaccard similarity coefficients.

Following group identification, differences between clusters were assessed using one-way ANOVA for normally distributed continuous variables and the Kruskal–Wallis test for non-normally distributed continuous variables. When the global Kruskal–Wallis test indicated between-cluster differences, pairwise comparisons were performed using the Dwass–Steel–Critchlow–Fligner (DSCF) procedure, which accounts for multiple comparisons. For variables analyzed using ANOVA, no post-hoc pairwise comparisons were required because no significant global between-cluster differences were observed. For non-parametric analyses, effect sizes were estimated using epsilon-squared (ε²) to quantify the magnitude of between-cluster differences.

A *p*-value < 0.05 was considered statistically significant. The analyses were performed using JAMOVI (version 2.3.28) using the snowCluster module for hierarchical clustering procedures or RStudio (version 2025.5.1) using the clusterboot function from the fpc package.

## Results

### Characteristics of the study population

The baseline clinical, metabolic, and inflammatory characteristics of the overall population are presented in Table 1. A total of 132 elderly women with a mean age of 70.8 ± 6.8 years were included in the study. Their mean BMI was 29.6 ± 5.0 kg/m², and the median waist circumference was 93.0 cm, indicating that most participants were overweight or obese. The median HOMA-IR was 2.12 (IQR: 1.34–3.48), and median vitamin D levels were 19.6 ng/mL (IQR: 14.2–25.1), with a high prevalence (57.6%) of vitamin D deficiency (<20 ng/mL).

**Table 1:** Baseline clinical, metabolic, and inflammatory characteristics of the study population

Variable	Total Population (*n* = 132)
**Demographic Characteristics**	
Age (years)	70.8 ± 6.8
Time since menopause (years)	23.5 ± 8.4
Menopause age (years)	47.2 ± 5.7
Hormone therapy use, n (%)	12 (9.1)
Physically active, n (%)	37 (28.0)
Diabetes mellitus, n (%)	22 (16.7)
Hypertension, n (%)	80 (60.6)
Continuous medication use, n (%)	101(76.5)
**Anthropometry**	
Weight (kg)	71.9 ± 12.8
Height (m)	1.55 ± 0.07
BMI (kg/m²)	29.6 ± 5.0
Waist circumference (cm)	93.0 (86.0–101.0)
**Metabolic Profile**	
Vitamin D (ng/mL)	19.6 (14.2–25.1)
Fasting glucose (mg/dL)	92.2 ± 10.3
Insulin (µU/mL)	9.0 (5.7–14.8)
HOMA-IR	2.12 (1.34–3.48)
HbA1c (%)	5.13 ± 0.58
Alkaline phosphatase (U/L)	92.6 ± 27.8
PTH (pg/mL)	60.9 ± 22.4
Creatinine (mg/dL)	0.74 ± 0.15
Calcium (mg/dL)	9.62 ± 0.54
**Hemodynamics**	
SBP (mmHg)	135.3 ± 20.0
DBP (mmHg)	80.7 ± 12.0
**Lipid Profile**	
Triglycerides (mg/dL)	158.0 (101.0–211.5)
HDL-c (mg/dL)	51.8 ± 11.3
LDL (mg/dL)	125.8 ± 37.6
Total Cholesterol (mg/dL)	208.2 ± 43.6
**Inflammatory Profile**	
IL-6 (pg/mL)	1.10 (0.40–2.80)
TNF-α (pg/mL)	10.00 (6.80–13.80)
IL-10 (pg/mL)	2.90 (0.50–4.90)

Values are presented as mean ± standard deviation for normally distributed variables, median (interquartile range) for non-normally distributed variables, or absolute frequency (percentage) for categorical variables

*BMI* body mass index, *HOMA-IR* homeostasis model assessment of insulin resistance, *HbA1c* glycated hemoglobin, *PTH* parathyroid hormone, *SBP* systolic blood pressure, *DBP* diastolic blood pressure, *HDL-c* high-density lipoprotein cholesterol, *LDL-c* low-density lipoprotein cholesterol, *IL-6* interleukin-6, *TNF-α* tumor necrosis factor-alpha and IL-10 interleukin-10

### Identification of immunometabolic clusters

Unsupervised hierarchical cluster analysis based on metabolic and inflammatory variables identified three data-driven immunometabolic profiles. The silhouette coefficient was highest for three-cluster solution (0.408), compared with two (0.246) and four clusters (0.332). Bootstrap resampling indicated moderate internal cluster stability, with mean Jaccard similarity coefficients of 0.699 for Cluster 1, 0.619 for Cluster 2, and 0.646 for Cluster 3. These values suggest that the three-cluster solution captured moderately stable group-level patterns under resampling, although uncertainty remains regarding individual-level cluster assignment.

No significant differences in age (*p* = 0.913) or BMI (*p* = 0.491) were observed across the identified profiles. The complete clinical, metabolic, and inflammatory characteristics of each profile are presented in Table 2. The three profiles comprised 25, 50, and 57 participants, respectively. Cluster 1 was characterized by lower IL-10 concentrations and a less favorable lipid profile, while systolic and diastolic blood pressure were similarly elevated in Clusters 1 and 2 relative to Cluster 3. Cluster 2 showed an overall intermediate profile, with intermediate fasting glucose and IL-10 values, while its lipid profile was similar to Cluster 3. Its most distinctive feature was the lowest vitamin D concentration among the three clusters. Cluster 3 was characterized by lower blood pressure, higher IL-10 concentrations, and a more favorable lipid profile.

**Table 2:** Clinical, metabolic, inflammatory, and lipid profile characteristics according to clusters

Variable	Cluster 1 (*n* = 25)	Cluster 2 (*n* = 50)	Cluster 3 (*n* = 57)	*p*-value		ε²
**Demographics**						
Age (years)	68.00 (66.00–73.00)	69.50 (66.00–75.00)	69.00 (66.00–76.00)	0.9132	0.0014	
Time since menopause (years)	23.00 (16.00–30.00)	19.50 (16.25–27.75)	21.00 (17.00–31.00)	0.8916	0.0018	
Hormone therapy use, n (%)	4 (16.0)	4 (8.0)	4 (7.0)	0.441^*^	-	
Physically active, n (%)	9 (36.0)	12 (24.0)	16 (28.1)	0.552	-	
Hypertension, n (%)	19 (76.0)	31 (62.0)	30 (52.6)	0.133	-	
Diabetes mellitus, n (%)	6 (24.0)	9 (18.0)	7 (12.3)	0.402	-	
Continuous medication use, n (%)	20 (80.0)	37 (74.0)	44 (77.2)	0.835	-	
**Anthropometry**						
Weight (kg)	70.60 ± 12.65	73.46 ± 11.59	71.65 ± 13.68	0.5211	-	
Height (m)	1.55 ± 0.07	1.56 ± 0.06	1.56 ± 0.07	0.9049	-	
BMI (kg/m²)	30.41 (26.02–32.44)	30.62 (26.95–33.18)	29.21 (25.35–32.41)	0.4913	0.0108	
Waist Circumference (cm)	96.00 (91.00–102.00)	95.00 (87.25–101.00)	89.00 (85.00–101.00)	0.2025	0.0244	
**Metabolic Profile**						
Vitamin D (ng/mL)	18.60 (13.80–25.40)ᵃ	14.75 (11.60–20.50)ᵇ	21.30 (15.30–28.00)ᵃ	**0.0010**	**0.1062**	
Fasting Glucose (mg/dL)	98.00 (91.00–107.00)ᵃ	92.00 (88.00–98.75)ᵇ	87.00 (81.00–95.00)ᶜ	**0.0001**	**0.1449**	
Insulin (µU/mL)	9.90 (8.10–13.80)ᵃ	9.90 (7.15–14.93)ᵃ	8.00 (5.40–12.40)ᵃ	**0.0436**	**0.0478**	
HOMA-IR	2.74 (1.86–3.48)ᵃ	2.27 (1.51–3.24)ᵃ	1.70 (1.12–3.00)ᶜ	**0.0109**†	**0.0690**	
HbA1c (%)	5.44 (5.06–5.94)ᵃ	5.11 (4.89–5.49)ᵇ	4.83 (4.50–5.28)ᶜ	**0.0001**†	**0.1449**	
Alkaline Phosphatase (U/L)	79.00 (65.00–98.00)	78.00 (65.00–97.00)	86.00 (71.00–111.00)	0.0856	0.0375	
PTH (pg/mL)	54.30 (42.30–76.80)	57.00 (42.00–76.50)	56.40 (42.50–78.90)	0.8921	0.0017	
Creatinine (mg/dL)	0.70 (0.60–0.80)	0.70 (0.60–0.80)	0.70 (0.60–0.90)	0.7654	0.0042	
Calcium (mg/dL)	9.40 (9.20–9.80)	9.40 (9.20–9.70)	9.50 (9.30–9.90)	0.2345	0.0221	
**Inflammatory Profile**						
IL-6 (pg/mL)	0.73 (0.35–1.40)	1.31 (0.65–2.08)	1.08 (0.52–1.76)	0.0757	0.0394	
TNF-α (pg/mL)	10.11 (5.33–14.37)ᵃ	9.15 (6.52–10.89)ᵃ	11.37 (8.34–15.98)ᵃ	**0.0095**	**0.0710**	
IL-10 (pg/mL)	0.05 (0.03–0.10)ᵃ	1.15 (0.22–3.03)ᵇ	3.20 (1.80–7.32)ᶜ	**<0.001**	**0.4159**	
**Hemodynamics**						
SBP (mmHg)	142.00 (135.00–160.00)ᵃ	144.50 (135.25–159.75)ᵃ	121.00 (112.00–129.00)ᶜ	**<0.001**	**0.4848**	
DBP (mmHg)	87.00 (82.00–93.00)ᵃ	86.00 (77.50–92.00)ᵃ	73.00 (68.00–80.00)ᶜ	**<0.001**	**0.3760**	
**Lipid Profile**						
Triglycerides (mg/dL)	243.00 (178.00–311.00)ᵃ	124.50 (93.50–165.25)ᵇ	128.00 (98.00–174.00)ᵇ	**<0.001**	**0.2564**	
HDL-c (mg/dL)	48.00 (41.00–50.00)ᵃ	52.50 (47.25–60.00)ᵇ	52.00 (44.00–61.00)ᵇ	**0.0042**	**0.0837**	
LDL-c (mg/dL)	125.40 (99.60–172.80)	122.60 (99.40–148.20)	126.40 (105.60–156.20)	0.9663	0.0005	
Total Cholesterol (mg/dL)	227.00 (193.00–274.00)ᵃ	198.00 (156.25–217.00)ᵇ	209.00 (183.00–227.00)ᵇ	**0.0049**	**0.0811**	

Values are presented as mean ± standard deviation (SD) for normally distributed variables and median (interquartile range [IQR]) for non-normally distributed variables. Between-group comparisons were performed using one-way ANOVA for normally distributed variables and the Kruskal–Wallis test for non-normally distributed variables. Pairwise comparisons following significant Kruskal–Wallis tests were performed using the Dwass–Steel–Critchlow–Fligner (DSCF) procedure. Effect sizes for non-parametric comparisons are reported as epsilon squared (ε²)

*BMI* body mass index, *PTH* parathyroid hormone, *HOMA-IR* homeostasis model assessment of insulin resistance, *HbA1c* glycated hemoglobin, *IL-6* interleukin-6, *IL-10* interleukin-10, *TNF-α* tumor necrosis factor alpha, *SBP* systolic blood pressure, *DBP* diastolic blood pressure, *HDL-c* high-density lipoprotein cholesterol, *LDL-c* low-density lipoprotein cholesterol

For hormone therapy use, ^*^Fisher’s exact test

†Detailed DSCF pairwise comparisons are presented in Table 3

Different superscript letters (ᵃ, ᵇ, ᶜ) within the same row indicate statistically significant pairwise differences between clusters (*p* < 0.05), whereas clusters sharing the same letter are not significantly different. Variables without superscript letters did not present significant pairwise differences

Although the global Kruskal–Wallis tests were significant for insulin (*p* = 0.0436) and TNF-α (*p* = 0.0095), no pairwise comparison remained statistically significant after DSCF post-hoc testing; therefore, all clusters share the same superscript letter (ᵃ) for these variables

### Key differentiating markers between clusters

The variables showing the largest between-cluster effect sizes (ε²) were SBP (ε² = 0.4848) and IL-10 (ε² = 0.4159), both showing very large effects.

Cluster 1 also exhibited a less favorable lipid profile, with higher triglyceride and total cholesterol concentrations and lower HDL-c levels than Clusters 2 and 3 (*p* < 0.05). In addition, vitamin D concentrations differed significantly across the identified profiles.

HbA1c and HOMA-IR were not directly included as clustering variables. HbA1c was external to cluster derivation, whereas HOMA-IR was not fully independent because fasting glucose, which contributes to its calculation, was included in the clustering procedure. HbA1c levels differed significantly across all three clusters (*p* < 0.05 for all pairwise comparisons), showing a graded pattern (Cluster 3 < Cluster 2 < Cluster 1). By contrast, HOMA-IR showed less between-cluster separation, with no significant difference between Clusters 1 and 2 (*p* = 0.704). Because fasting glucose was included in cluster derivation and contributes to HOMA-IR calculation, these between-cluster HOMA-IR differences should not be interpreted as fully independent external associations. Detailed overall and pairwise comparisons of the clinical, metabolic, and inflammatory variables across the three clusters are presented in Table 3.

**Table 3:** Pairwise comparisons of metabolic variables across clusters using the Dwass–Steel–Critchlow–Fligner (DSCF) test

Variable	Global ε²	Comparison	DSCF W	*p*-value
HOMA-IR	0.0690	Cluster 1 vs Cluster 2	−1.1286	0.704
		Cluster 1 vs Cluster 3	−3.5970	0.029^*^
		Cluster 2 vs Cluster 3	−3.3821	0.044^*^
HbA1c	0.1449	Cluster 1 vs Cluster 2	−3.5105	0.034^*^
		Cluster 1 vs Cluster 3	−5.7949	<0.001^***^
		Cluster 2 vs Cluster 3	−3.5189	0.034^*^

Pairwise comparisons were conducted using the DSCF multiple comparison procedure following significant overall differences detected by the Kruskal–Wallis test

*HOMA-IR* homeostasis model assessment of insulin resistance, *HbA1c* glycated hemoglobin

The reported ε² values correspond to the effect sizes obtained from the overall Kruskal–Wallis tests for each variable. Significance levels: *p < 0.05; ***p < 0.001

**Fig. 1:**
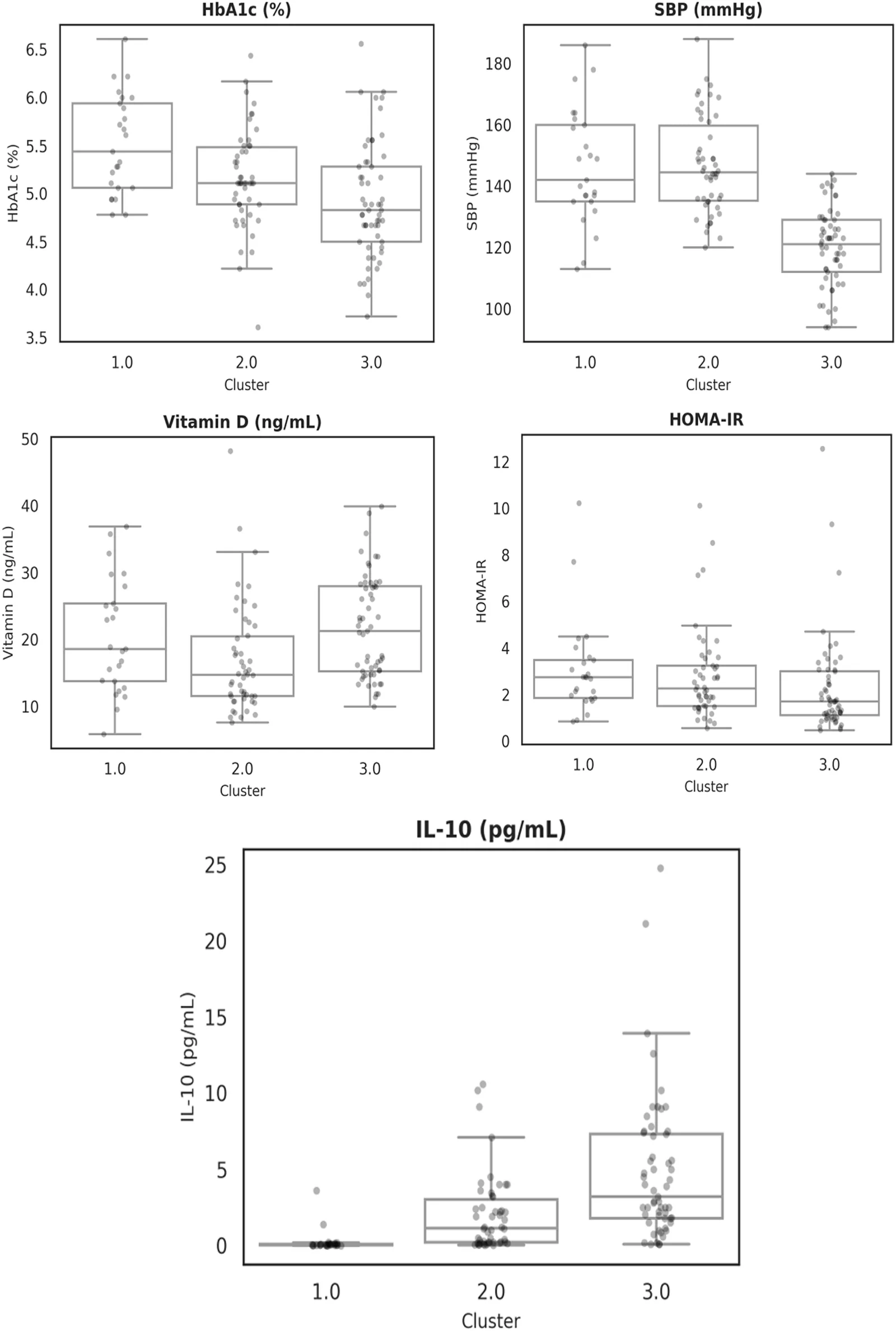
Distribution of key metabolic, inflammatory, and hemodynamic markers across clusters. Boxplots show median and interquartile range with individual observations overlaid. Significant between-cluster differences were observed for IL-10 (ε² = 0.4159), systolic blood pressure (ε² = 0.4848), HbA1c (ε² = 0.1449), vitamin D (ε² = 0.1062), and HOMA-IR (ε² = 0.0690). Effect sizes are reported as epsilon-squared (ε²)

The distribution and comparison of these key markers (HbA1c, SBP, vitamin D, HOMA-IR, and IL-10) across the three clusters are visually represented in Fig. 1.

### Sensitivity analyses of cluster solution

Sensitivity analyses indicated that the original three-cluster solution was influenced by key discriminating variables. In the original model, the three-cluster solution showed the highest silhouette coefficient (0.408), whereas exclusion of SBP reduced the silhouette coefficient for three clusters to 0.133 and resulted in a higher silhouette value for a two-cluster solution (0.433). Similarly, exclusion of IL-10 reduced separation among clusters (three-cluster silhouette = 0.148). Removal of both SBP and IL-10 resulted in low silhouette coefficients across all tested solutions (0.122–0.134). Exclusion of fasting glucose also altered the clustering structure, with a two-cluster solution showing the highest silhouette coefficient (0.435). Bootstrap stability coefficients for the sensitivity models are presented in Supplementary Table 1.

## Discussion

The present study identified three data-driven immunometabolic profiles in elderly postmenopausal women, indicating substantial heterogeneity in the way metabolic, inflammatory, and hemodynamic characteristics coexist within this population. The profiles differed across several of these variables, with one group exhibiting a more favorable profile and another showing a less favorable combination of these markers. Importantly, the profiles also showed distinct patterns in the additional glycemic measures examined. HbA1c exhibited a graded pattern across clusters, whereas HOMA-IR did not consistently distinguish all groups. These findings indicate that HbA1c showed a more clearly graded pattern across the identified immunometabolic profiles than HOMA-IR. However, because fasting glucose was included in cluster derivation and also contributes to HOMA-IR calculation, the between-cluster differences in HOMA-IR should not be interpreted as fully independent of the cluster structure. Collectively, these findings indicate that immunometabolic heterogeneity in late postmenopausal women is not fully captured by single markers and may be better understood through analysis of integrated multivariate profiles.

The present findings illustrate how combinations of inflammatory, metabolic, and hemodynamic markers can be organized into data-driven multivariate profiles within this cohort. Traditional analytical strategies typically examine these variables independently, which may obscure underlying structure in the data. In contrast, the clustering approach considers the joint configuration of multiple variables, allowing the identification of profiles that may not be apparent from single-marker analyses. This perspective aligns with emerging evidence that complex physiological states, particularly in aging, may be more comprehensively characterized through multivariate frameworks than through single-marker approaches [6, 18, 19].

SBP showed the largest between-cluster effect size (ε² = 0.4848), followed by IL-10 (ε² = 0.4159). Higher SBP characterized both Clusters 1 and 2 relative to Cluster 3, whereas Cluster 3 combined lower blood pressure with the highest IL-10 concentrations and relatively higher vitamin D concentrations than Cluster 2. This configuration is biologically compatible with evidence linking anti-inflammatory regulatory pathways and vitamin D-related mechanisms to vascular homeostasis [25–27]. Notably, Cluster 3 also exhibited the lowest HbA1c concentration, a glycemic marker not included in cluster derivation. Thus, the profile characterized by lower blood pressure, higher IL-10, and relatively higher vitamin D than Cluster 2 was also associated with lower chronic glycemic exposure [28], suggesting that these hemodynamic, inflammatory, vitamin D-related, and glycemic characteristics may coexist within a broader immunometabolic pattern. However, because SBP, IL-10, and vitamin D contributed to cluster derivation and the study was cross-sectional, these findings should be interpreted as an integrated pattern of co-occurring characteristics rather than as evidence of causal or temporal relationships.

Although TNF-α showed a significant global difference across clusters, post-hoc analyses did not identify significant pairwise contrasts after correction for multiple testing. In contrast, IL-10 exhibited a robust and progressive gradient and represented one of the main variables structuring the cluster solution. Because IL-10 was included in the clustering procedure, this finding should be interpreted as part of the cluster-defining pattern rather than as independent validation of the profiles. Biologically, the pronounced IL-10 gradient, together with the absence of significant pairwise differences in TNF-α, suggests that anti-inflammatory regulation represented a more prominent inflammatory dimension of the identified profiles in this cohort. This interpretation is compatible with the concept of inflammaging, whereby chronic low-grade inflammatory activation coexists with counter-regulatory mechanisms that preserve immune homeostasis and metabolic adaptation during aging [9, 29].

Sensitivity analyses provided additional insight into the dependence of the cluster solution on key discriminating variables. The marked reduction in cluster separation after removing SBP, IL-10, or both indicates that the primary three-cluster solution was substantially structured by hemodynamic and anti-inflammatory regulatory dimensions. Similarly, exclusion of fasting glucose altered the clustering structure, highlighting the contribution of glycemic regulation to the identified profiles and reinforcing the need for cautious interpretation of HOMA-IR, given its mathematical dependence on fasting glucose. Therefore, the identified clusters should not be interpreted as stable phenotypes independent of these variables, but rather as exploratory immunometabolic profiles in which SBP, IL-10, and fasting glucose play central organizing roles within the broader multivariate structure.

A notable finding of this study was the different pattern observed for HOMA-IR and HbA1c across the cluster-derived profiles. In women more than two decades after menopause, HOMA-IR showed less separation between Cluster 2 and Cluster 1, with no significant difference between these two data-driven profiles. The lower between-profile separation observed for HOMA-IR may, in part, be related to age-associated changes in insulin dynamics that can affect the interpretation of fasting-based indices in older adults [5]. In contrast, HbA1c exhibited a more graded pattern across the identified immunometabolic profiles, consistent with its representation of longer-term glycemic exposure rather than fasting-state insulin resistance alone [6, 7]. HbA1c showed a progressive increase across the three profiles (Cluster 3 < Cluster 2 < Cluster 1; *p* < 0.05 for all pairwise comparisons). This more graded pattern is consistent with the different physiological information provided by HbA1c, which reflects longer-term glycemic exposure, compared with HOMA-IR, which is derived from fasting glucose and insulin [7, 19]. Moreover, previous studies have demonstrated that optimal HOMA-IR thresholds vary according to age and sex, particularly among women, suggesting that age-specific reference values may be more appropriate in older populations [30]. Accordingly, the HOMA-IR results presented herein should be interpreted as continuous indicators of metabolic status rather than as definitive diagnostic classifications of insulin resistance.

Cluster 1 represented the least favorable overall immunometabolic profile within this cohort, characterized by higher glycemic values, a less favorable lipid profile, and the lowest IL-10 concentrations. In contrast, Cluster 2 showed an intermediate immunometabolic profile, although the individual variables did not uniformly follow an intermediate pattern. Fasting glucose and IL-10 showed intermediate values, whereas blood pressure was similar to Cluster 1 and the lipid profile was similar to Cluster 3. Within this mixed multivariate pattern, the lower vitamin D concentration emerged as the most distinctive characteristic of Cluster 2. This finding should be interpreted as a distinctive feature of the intermediate Cluster 2 rather than as evidence that this profile was more adverse than Cluster 1 or represented an earlier stage of immunometabolic deterioration.

Notably, median vitamin D concentrations in all three clusters remained below the conventional 30 ng/mL threshold for vitamin D sufficiency [2], indicating that the cluster central values were within a restricted spectrum ranging from deficiency to insufficiency. Therefore, the differences observed between clusters should be interpreted as relative variations within an overall suboptimal vitamin D spectrum rather than as comparisons between deficient and sufficient vitamin D states. Accordingly, the lower vitamin D concentration observed in Cluster 2 should not be interpreted as a categorical indication of greater clinical severity.

Although Cluster 1 represented the most unfavorable overall immunometabolic profile, its median vitamin D concentration was higher than that observed in Cluster 2. Rather than representing a discrepancy, this finding reinforces that the cluster-derived profiles reflect different multivariate combinations and should not be interpreted as an ordered continuum of clinical severity. Even within this overall suboptimal vitamin D spectrum, relative differences in vitamin D formed part of the multivariate structure distinguishing the derived profiles. Overall, these observations suggest that vitamin D status may be one component of the broader multivariate pattern linking metabolic, inflammatory, and hemodynamic characteristics in older women [11, 31]. Prospective studies are needed to clarify the temporal relationship between vitamin D status and changes in immunometabolic profiles during aging [32].

Longitudinal studies in other populations have suggested that metabolic risk clusters can show considerable persistence over time [21]. However, the stability of the specific immunometabolic profiles identified in this study requires direct longitudinal evaluation. Studies in other populations suggest that multivariate metabolic profiles may capture aspects of metabolic vulnerability that are not fully represented by anthropometric measures alone [20, 33]. Some of these profiles have also been associated with clinically relevant outcomes, including all-cause mortality [20]. However, such findings cannot be extrapolated directly to the exploratory profiles identified in the present study and do not establish their clinical or prognostic validity.

From a clinical research perspective, the identification of distinct immunometabolic profiles may help characterize the considerable heterogeneity observed among elderly postmenopausal women. The menopausal transition is increasingly recognized as a cardiometabolic turning point, involving complex interactions among adiposity, inflammation, vascular function, and glucose homeostasis that do not evolve uniformly across individuals [34, 35]. Within this framework, multidimensional profiling approaches may provide a broader characterization of metabolic heterogeneity than approaches focused on isolated biomarkers [6, 18, 19]. Nevertheless, given the cross-sectional nature of the present study, these implications should be interpreted cautiously, and prospective investigations are needed to determine whether the identified profiles possess predictive value for clinically relevant outcomes.

We acknowledge the limitations inherent in the cross-sectional design, which does not permit causal or temporal inferences regarding the relationships among vitamin D, IL-10, and glycemic status. Furthermore, visceral adiposity was not directly assessed and therefore remains a potential source of residual confounding. Similar BMI values across clusters do not exclude differences in fat distribution, particularly visceral fat, which has been associated with lower circulating vitamin D concentrations and increased production of pro-inflammatory mediators [1]. Such differences could have influenced the observed immunometabolic patterns independently of overall adiposity. An additional limitation is that medication use was recorded only as a dichotomous variable (continuous medication use: yes/no), without specification of individual pharmacological classes. Consequently, it was not possible to evaluate the potential influence of antidiabetic agents, antihypertensive drugs, or statins on metabolic and inflammatory biomarkers. Although the prevalence of diabetes, hypertension, and overall medication use did not differ significantly across clusters, residual confounding related to specific treatments cannot be completely excluded. Therefore, similar overall medication use across clusters does not exclude differential effects of specific pharmacological classes on the observed metabolic and inflammatory patterns. Although bootstrap resampling supported the presence of moderately stable cluster structures, the Jaccard coefficients indicate that the identified profiles should be interpreted as exploratory group-level patterns rather than definitive or externally validated phenotypes. Finally, the absence of data on sun exposure and dietary vitamin D intake limits a more comprehensive assessment of factors that may have influenced vitamin D status in this cohort.

## Conclusion

In this exploratory cross-sectional analysis, three data-driven immunometabolic profiles were identified based on integrated immunometabolic patterns rather than isolated markers. The graded differences in HbA1c across the identified profiles suggest that longer-term glycemic exposure may represent an additional dimension of immunometabolic heterogeneity in older postmenopausal women. These profiles should be interpreted as exploratory groupings rather than definitive clinical phenotypes.

## Supplementary information

DATA SUPPLEMENTS

## Data Availability

The data that support the findings of this study are available from the corresponding author upon reasonable request.

## References

[1] J. Abildgaard, T. Ploug, E. Al-Saoudi, T. Wagner, C. Thomsen, C. Ewertsen, M. Bzorek, B.K. Pedersen, A.T. Pedersen, B. Lindegaard, Changes in abdominal subcutaneous adipose tissue phenotype following menopause is associated with increased visceral fat mass. Sci. Rep. **11**, 14750 (2021). 10.1038/s41598-021-94189-2 34285301 PMC8292317

[2] P. Anagnostis, S. Livadas, D.G. Goulis, S. Bretz, I. Ceausu, F. Durmusoglu, R. Erkkola, I. Fistonic, M. Gambacciani, M. Geukes, H. Hamoda, C. Hartley, A.L. Hirschberg, B. Meczekalski, N. Mendoza, A. Mueck, A. Smetnik, P. Stute, M. van Trotsenburg, M. Rees, I. Lambrinoudaki, EMAS position statement: vitamin D and menopausal health. Maturitas **169**, 2–9 (2023). 10.1016/j.maturitas.2022.12.006 36566517

[3] S. Fong, K. Pabis, D. Latumalea, N. Dugersuren, M. Unfried, N. Tolwinski, B. Kennedy, J. Gruber, Principal component-based clinical aging clocks identify signatures of healthy aging and targets for clinical intervention. Nat. Aging **4**, 1137–1152 (2024). 10.1038/s43587-024-00646-8 38898237 PMC11333290

[4] X. Shen, C. Wang, X. Zhou, W. Zhou, D. Hornburg, S. Wu, M.P. Snyder, Nonlinear dynamics of multi-omics profiles during human aging. Nat. Aging **4**, 1619–1634 (2024). 10.1038/s43587-024-00692-2 39143318 PMC11564093

[5] O.P. Zaharia, K. Strassburger, A. Strom, G.J. Bönhof, Y. Karusheva, S. Antoniou, K. Bódis, D.F. Markgraf, V. Burkart, K. Müssig, J.-H. Hwang, O. Asplund, L. Groop, E. Ahlqvist, J. Seissler, P. Nawroth, S. Kopf, S.M. Schmid, M. Stumvoll, A.F.H. Pfeiffer, S. Kabisch, S. Tselmin, H.U. Häring, D. Ziegler, O. Kuss, J. Szendroedi, M. Roden, B.-F. Belgardt, A. Buyken, J. Eckel, G. Geerling, H. Al-Hasani, C. Herder, J.-H. Hwang, A. Icks, J. Kotzka, O. Kuss, E. Lammert, D. Markgraf, K. Müssig, W. Rathmann, M. Roden, J. Szendroedi, D. Ziegler, Risk of diabetes-associated diseases in subgroups of patients with recent-onset diabetes: a 5-year follow-up study. Lancet Diabetes Endocrinol. **7**, 684–694 (2019). 10.1016/S2213-8587(19)30187-1 31345776

[6] R. González-Martos, J. Galeano, C. Ramirez-Castillejo, N. Gusi, E. Gesteiro, G. Vicente-Rodriguez, I. Ara, A. Guadalupe-Grau, Unsupervised clustering of biochemical markers reveals health profiles associated with function and survival in active aging. Sci. Rep. **15**, 30546 (2025). 10.1038/s41598-025-14580-1 40835865 PMC12368133

[7] G.S. Hotamisligil, Inflammation, metaflammation and immunometabolic disorders. Nature **542**, 177–185 (2017). 10.1038/nature21363 28179656

[8] P.C. Calder, N. Bosco, R. Bourdet-Sicard, L. Capuron, N. Delzenne, J. Doré, C. Franceschi, M.J. Lehtinen, T. Recker, S. Salvioli, F. Visioli, Health relevance of the modification of low grade inflammation in ageing (inflammageing) and the role of nutrition. Ageing Res. Rev. **40**, 95–119 (2017). 10.1016/j.arr.2017.09.001 28899766

[9] C. Franceschi, P. Garagnani, P. Parini, C. Giuliani, A. Santoro, Inflammaging: a new immune–metabolic viewpoint for age-related diseases. Nat. Rev. Endocrinol. **14**, 576–590 (2018). 10.1038/s41574-018-0059-4 30046148

[10] T.V. Rohm, D.T. Meier, J.M. Olefsky, M.Y. Donath, Inflammation in obesity, diabetes and related disorders. Immunity **55**, 31–55 (2022). 10.1016/j.immuni.2021.12.013 35021057 PMC8773457

[11] C. Fantini, C. Corinaldesi, A. Lenzi, S. Migliaccio, C. Crescioli, Vitamin D as a shield against aging. Int. J. Mol. Sci. **24**, 4546 (2023). 10.3390/ijms24054546 36901976 PMC10002864

[12] D.E. Martin, B.L. Torrance, L. Haynes, J.M. Bartley, Targeting aging: lessons learned from immunometabolism and cellular senescence. Front. Immunol. **12**, 714742 (2021). 10.3389/fimmu.2021.714742 34367184 PMC8334863

[13] P. Ginefra, H.C. Hope, G. Lorusso, P. D’Amelio, N. Vannini, The immunometabolic roots of aging. Curr. Opin. Immunol. **91**, 102498 (2024). 10.1016/j.coi.2024.102498 39461330

[14] V.T. Samuel, G.I. Shulman, Mechanisms for insulin resistance: common threads and missing links. Cell **148**, 852–871 (2012). 10.1016/j.cell.2012.02.017 22385956 PMC3294420

[15] M. Li, X. Chi, Y. Wang, S. Setrerrahmane, W. Xie, H. Xu, Trends in insulin resistance: insights into mechanisms and therapeutic strategy. Signal. Transduct. Target. Ther. **7**, 216 (2022). 10.1038/s41392-022-01073-0 35794109 PMC9259665

[16] S.-H. Lee, S.-Y. Park, C.S. Choi, Insulin resistance: from mechanisms to therapeutic strategies. Diabetes Metab. J. **46**, 15–37 (2021). 10.4093/dmj.2021.0280 34965646 PMC8831809

[17] L. Trtica Majnarić, Z. Bosnić, T. Kurevija, T. Wittlinger, Cardiovascular risk and aging: the need for a more comprehensive understanding. J. Geriatr. Cardiol. **18**, 462–478 (2021). 10.11909/j.issn.1671-5411.2021.06.004 34220975 PMC8220387

[18] S. Galbraith, J.A. Daniel, B. Vissel, A study of clustered data and approaches to its analysis. J. Neurosci. **30**, 10601–10608 (2010). 10.1523/JNEUROSCI.0362-10.2010 20702692 PMC6634702

[19] Y. Aoki, B. Hamrén, L.E. Clegg, C. Stahre, D.L. Bhatt, I. Raz, B.M. Scirica, J. Oscarsson, B. Carlsson, Assessing reproducibility and utility of clustering of patients with type 2 diabetes and established CV disease (SAVOR -TIMI 53 trial). PLoS One **16**, e0259372 (2021). 10.1371/journal.pone.0259372 34797832 PMC8604302

[20] A. Higuera-Gómez, V. de la O, R. San-Cristobal, R. Ribot-Rodríguez, I. Espinosa-Salinas, A. Dávalos, M.P. Portillo, J.A. Martínez, Computational algorithm based on health and lifestyle traits to categorize lifemetabotypes in the NUTRiMDEA cohort. Sci. Rep. **14**, 24835 (2024). 10.1038/s41598-024-75110-z 39438551 PMC11496800

[21] Y.-J. Choi, G.S. Kim, Transitions in metabolic syndrome clustering patterns before and after menopause: a latent transition analysis in Korean women. Menopause **33**, 449 (2026). 10.1097/GME.0000000000002689 41364541

[22] M. Davies, G. Sarri, M.A. Lumsden, Diagnosis of the menopause: NICE guidance and quality standards. Ann. Clin. Biochem. **54**, 516–518 (2017). 10.1177/0004563217706381 28388870

[23] C.L. Orsatti, E.A.P. Nahas, J. Nahas-Neto, F.L. Orsatti, V.I. Giorgi, S.S. Witkin, Evaluation of toll-like receptor 2 and 4 RNA expression and the cytokine profile in postmenopausal women with metabolic syndrome. PLoS One **9**, e109259 (2014). 10.1371/journal.pone.0109259 25329057 PMC4201477

[24] B. Geloneze, A.C.J. Vasques, C.F.C. Stabe, J.C. Pareja, L.E.F.P.D.L. Rosado, E.C.D. Queiroz, M.A. Tambascia, HOMA1-IR and HOMA2-IR indexes in identifying insulin resistance and metabolic syndrome: Brazilian Metabolic Syndrome Study (BRAMS). Arq. Bras. Endocrinol. Metab. **53**, 281–287 (2009). 10.1590/S0004-27302009000200020 19466221

[25] Y.C. Li, G. Qiao, M. Uskokovic, W. Xiang, W. Zheng, J. Kong, Vitamin D: a negative endocrine regulator of the renin-angiotensin system and blood pressure. J. Steroid Biochem. Mol. Biol. **89–90**, 387–392 (2004). 10.1016/j.jsbmb.2004.03.004 15225806

[26] C. de Oliveira, J.P. Biddulph, V. Hirani, I.J.C. Schneider, Vitamin D and inflammatory markers: cross-sectional analyses using data from the English Longitudinal Study of Ageing (ELSA). J. Nutr. Sci. **6**, e1 (2017). 10.1017/jns.2016.37 28620476 PMC5465858

[27] S. Ramuth, R.L. Carvalho, R.Z. Moscogliato, M. Rossi, L.H.D.S. Nali, P. Colombo-Souza, J.B. Do Amaral, G.E. Furtado, T.R.P. de Brito, A.L.L. Bachi, New insights into the association between cardiometabolic index with metabolic profile, nutritional status, and inflammaging in older adults. Front. Aging **6**, 1699767 (2026). 10.3389/fragi.2025.1699767 41602164 PMC12833520

[28] American Diabetes Association Professional Practice Committee for Diabetes*: 6, Glycemic goals, hypoglycemia, and hyperglycemic crises: standards of care in diabetes—2026. Diabetes Care **49**, S132–S149 (2025). 10.2337/dc26-S006 PMC12690178 41358894

[29] A. Singh, S.H. Schurman, A. Bektas, M. Kaileh, R. Roy, D.M. Wilson, R. Sen, L. Ferrucci, Aging and inflammation. Cold Spring Harb. Perspect. Med. **14**, a041197 (2024). 10.1101/cshperspect.a041197 38052484 PMC11146314

[30] P. Gayoso-Diz, A. Otero-González, M.X. Rodriguez-Alvarez, F. Gude, F. García, A. De Francisco, A.G. Quintela, Insulin resistance (HOMA-IR) cut-off values and the metabolic syndrome in a general adult population: effect of gender and age: EPIRCE cross-sectional study. BMC Endocr. Disord. **13**, 47 (2013). 10.1186/1472-6823-13-47 24131857 PMC4016563

[31] H.R. Nematollahi, R. Hosseini, A. Bijani, H. Akhavan-Niaki, H. Parsian, M. Pouramir, M. Saravi, M. Bagherzadeh, A. Mosapour, M. Saleh-Moghaddam, M. Rajabian, M. Golpour, A. Mostafazadeh, Interleukin 10, lipid profile, vitamin D, selenium, metabolic syndrome, and serum antioxidant capacity in elderly people with and without cardiovascular disease: Amirkola health and ageing project cohort-based study. ARYA Atheroscler. **15**, 233–240 (2019). 10.22122/arya.v15i5.1623 31949450 PMC6954357

[32] E.S. Chambers, M. Vukmanovic-Stejic, C.T. Turner, B.B. Shih, H. Trahair, G. Pollara, E. Tsaliki, M. Rustin, T.C. Freeman, N.A. Mabbott, M. Noursadeghi, A.R. Martineau, A.N. Akbar, Vitamin D3 replacement enhances antigen-specific immunity in older adults. Immunother. Adv. **1**, ltaa008 (2021). 10.1093/immadv/ltaa008 36284901 PMC9585673

[33] S.H. Lim, T. Lee, J. Oh, K.-H. Hwang, E.H. Choi, S.-K. Cha, Waist circumference as an independent marker of insulin resistance: evidence from a nationwide Korean population study. J. Clin. Med. **14**, 7957 (2025). 10.3390/jcm14227957 41302993 PMC12653953

[34] R.E. Nappi, P. Chedraui, I. Lambrinoudaki, T. Simoncini, Menopause: a cardiometabolic transition. Lancet Diabetes Endocrinol. **10**, 442–456 (2022). 10.1016/S2213-8587(22)00076-6 35525259

[35] K.L. Marlatt, D.R. Pitynski‐Miller, K.M. Gavin, K.L. Moreau, E.L. Melanson, N. Santoro, W.M. Kohrt, Body composition and cardiometabolic health across the menopause transition. Obesity **30**, 14–27 (2022). 10.1002/oby.23289 34932890 PMC8972960

