# Supplementary material for: Cluster-derived immunometabolic profiles: association with glycemic dysregulation in elderly postmenopausal women
Source: Endocrine. 2026 Sep 9;91(1):290. doi: 10.1007/s12020-026-04762-w (PMC13558278; doi:10.1007/s12020-026-04762-w)
Supplement: Supplementary file 1 [file 12020_2026_4762_MOESM1_ESM.docx]

DATA SUPPLEMENTARY

**Dendrogram**

Supplementary Figure S1.

Dendrogram derived from the unsupervised hierarchical cluster analysis of participants based on standardized immunometabolic markers (Z-scores). Clustering was performed using Ward’s minimum variance method with squared Euclidean distances. The highlighted areas represent the three-cluster solution identified as optimal according to visual inspection of the dendrogram and the elbow method.

Supplementary Figure S2.

Heatmap of the immunometabolic markers included in the cluster analysis. Variables were standardized (Z-scores) to ensure equal weighting in cluster formation. Color intensity represents the relative magnitude of values (lighter tones indicate lower values and darker tones indicate higher values). The dendrogram on the left illustrates the hierarchical clustering of participants using Ward’s method based on squared Euclidean distances.

Supplementary Table S1. Summary of post-hoc pairwise comparison patterns for variables with significant overall differences

| **Variable** | **Cluster 1**  **(PMR)** | **Cluster 2**  **(TMP)** | **Cluster 3**  **(AIR)** | **Pattern of Differences** |
| --- | --- | --- | --- | --- |
| HOMA-IR | a | a | c | C3 differs from C1 and C2 |
| HbA1c (%) | a | b | c | All clusters differ (C1 > C2 > C3) |
| IL-10 (pg/mL) | a | b | c | All clusters differ (C1 < C2 < C3) |
| Fasting Glucose (mg/dL) | a | b | c | All clusters differ (C1 > C2 > C3) |
| Vitamin D (ng/mL) | a | b | a | C2 differs from C1 and C3 |
| Triglycerides (mg/dL) | a | b | b | C1 differs from C2 and C3 |
| HDL-c (mg/dL) | a | b | b | C1 differs from C2 and C3 |
| Total Cholesterol (mg/dL) | a | b | b | C1 differs from C2 and C3 |
| SBP (mmHg) | a | a | c | C3 differs from C1 and C2 |
| DBP (mmHg) | a | a | c | C3 differs from C1 and C2 |
| TNF-α (pg/mL) | a | a | a | No significant pairwise differences |
| Fasting Insulin (µU/mL) | a | a | a | No significant pairwise differences |

Overall Kruskal–Wallis test was significant (p < 0.05); however, post-hoc pairwise comparisons did not detect significant differences between clusters.

Supplementary Table S2. Sensitivity analyses evaluating the influence of selected variables on the clustering solution.

| **Sensitivity model** | **Variables excluded from clustering** | **Silhouette coefficient**  **(2 clusters)** | **Silhouette coefficient**  **(3 clusters)** | **Silhouette coefficient**  **(4 clusters)** | **Jaccard similarity coefficients**  **(3 clusters)** |
| --- | --- | --- | --- | --- | --- |
| Original model | None | 0.246 | **0.408** | 0.332 | 0.699, 0.619, 0.646 |
| Model 1 | Systolic blood pressure (SBP) | 0.433 | 0.133 | 0.126 | 0.689, 0.519, 0.712 |
| Model 2 | IL-10 | 0.129 | 0.148 | 0.115 | 0.723, 0.560, 0.686 |
| Model 3 | SBP + IL-10 | 0.122 | 0.134 | 0.125 | 0.712, 0.631, 0.755 |
| Model 4 | Fasting glucose | 0.435 | 0.119 | 0.115 | 0.673, 0.474, 0.653 |

Values represent mean silhouette coefficients for each tested cluster solution. Jaccard coefficients represent bootstrap-derived stability estimates for Clusters 1, 2, and 3, respectively (1000 resampling iterations). The original three-cluster solution showed the highest internal separation (silhouette coefficient = 0.408).

**Supplementary Figure S1. Distribution of clinical, metabolic, inflammatory, and lipid markers across clusters.**

Boxplots display median and interquartile range with individual data points overlaid. Between-group comparisons were performed using the Kruskal–Wallis test or one-way ANOVA, as appropriate. Significant overall differences were observed for vitamin D, fasting glucose, HbA1c, HOMA-IR, IL-10, systolic blood pressure (SBP), diastolic blood pressure (DBP), triglycerides, HDL-c, total cholesterol, fasting insulin, and TNF-α (p < 0.05).

Effect sizes (ε²) indicate very large effects for SBP (0.4848) and IL-10 (0.4159); large effects for triglycerides (0.2564), DBP (0.3760), fasting glucose (0.1449), and HbA1c (0.1449); moderate effects for vitamin D (0.1062), HDL-c (0.0837), total cholesterol (0.0811), HOMA-IR (0.0690), and TNF-α (0.0710); and small effects for fasting insulin (0.0478).
